# Organic or conventional production system and nutrient rate affect the nematode community in carrot production

**DOI:** 10.21307/jofnem-2021-082

**Published:** 2021-10-13

**Authors:** Zane J. Grabau, Danielle D. Treadwell, Jose J. Perez Orozco, David N. Campbell, Robert C. Hochmuth

**Affiliations:** 1 Entomology and Nematology Department, University of Florida, 1881 Natural Area Drive, Gainesville, FL, 32611; 2 Department of Horticultural Sciences, University of Florida, PO Box 110690, Gainesville, FL, 32611; 3 North Florida Research and Education Center-Suwannee Valley, University of Florida, 7580, CR 136, Live Oak, FL, 32060

**Keywords:** Carrot, *Daucus carota*, Organic, Management, Nutrient rate, Nutrient source, Nematode community

## Abstract

Organic and conventional production are common in horticulture crops and each system may exert a different influence on the soil ecosystem, particularly the nematode community. Crop nutrient rate is an important choice in both production systems. The objectives of this study were to assess the impacts of (i) organic and conventional production systems and (ii) nutrient rate in both systems on the nematode community in carrot production. To investigate these objectives, field studies in organic and conventional production – which included fumigation with 1,3-dichloropropene – were conducted in North-Central Florida. In both production systems, nutrient rate treatments were 168, 224, 280, 336, and 392 kg N/ha. Poultry litter was the nitrogen source in organic production whereas synthetic, inorganic fertilizer was used in conventional production. All nematode trophic groups were consistently more abundant in organic than conventional production. The nematode community was more diverse and had greater trophic structure in organic production. Greater rates of organic nutrients increased enrichment opportunists (bacterivores and fungivores), but inconsistently across years. Conventional production had similar results except that only moderate nutrient rates increased fungivore abundances. Extreme enrichment opportunists (*Rhabditis* spp.) drove bacterivore trends in organic production whereas moderate enrichment opportunists (*Cephalobus* spp.) drove trends in conventional production. Nutrient rates did not affect omnivore-predators, herbivores, nematode community diversity, or structure in either system. In summary, type of production system, organic or conventional, exerts a strong influence on the nematode community, but nutrient rate has less consistent effects in horticulture production.

Carrot (*Daucus carota* spp. *sativus*) production is an important industry in the United States, planted to 28,207 ha with a total crop value of $716 million (USD) in 2020 (NASS-USDA, 2021). Carrot production in Florida and the Southeast is a relatively small portion of that industry – undisclosed in production reports to protect privacy of individual growers (NASS-USDA, 2021). However, carrot production in the Southeast is growing, in part because it is strategically located near eastern US population centers. In Florida, organic production is an important section of the carrot industry as packinghouse demand outstrips production. In the United States, to be labelled organic, crops must be produced according to extensive and vigorous standards that are codified in law (United States Code, 2000). This is governed by the United States Department of Agriculture (USDA) and must be verified by a USDA-accredited certifying agent. Organic production must avoid prohibited materials such as most synthetic fertilizers or pesticides, genetically engineered plants, and various other external inputs. Additionally, maintaining soil fertility and pest management must be accomplished primarily by cultural or mechanical means with approved external materials used to supplement these methods. In sum, the intent of organic production is to ‘integrate cultural, biological, and mechanical practices that foster cycling of resources, promote ecological balance, and conserve biodiversity’, as specified by the USDA (United States Code, 2000). Comparing conventional to organic production is one method of assessing the efficacy of organic production at achieving this.

Soil health is an important aspect of the ecological balance and biodiversity that organic production seeks to improve. Soil health is defined by the USDA National Resources Conservation Service as ‘the continued capacity of soil to function as a vital living ecosystem that sustains plants, animals, and humans’ (USDA-National Resource Conservation Services, 2020). The nematode community is an important biological component of soil health because it can contribute to sustained soil productivity and can be a useful indicator of agroecosystem status (Grabau et al., 2020; Trap et al., 2016). The nematode community includes both plant-parasitic nematodes that parasitize and damage crops and free-living nematodes, which may be beneficial to soil productivity (Ferris et al., 2001; Grabau et al., 2018). Free-living nematodes include a wide range of trophic groups – including fungal-feeders, bacteria-feeders, predators, and omnivores (Ferris et al., 2001; Yeates et al., 1993). They can contribute directly to soil productivity through services such as pest suppression (Khan and Kim, 2005), nutrient cycling (Holajjer et al., 2016; Trap et al., 2016), and redistribution of microbes in the soil profile (Jiang et al., 2018). They can also serve as bioindicators of the broader soil ecosystem since they span a range of trophic groups and ecological niches or life strategies (Bongers, 1990; Yeates et al., 1993). The nematode community is known to be sensitive to many agricultural practices such as pesticide application (Grabau et al., 2020; Watson and Desaeger, 2019), tillage (Grabau et al., 2018; Neher et al., 2019), and crop rotation (Grabau and Chen, 2016), so it could be useful for assessing impacts of organic and conventional systems on the soil community.

The impacts of production system (organic or conventional) on the nematode community have been investigated in various crops and locations. Often, segments of the nematode community are more robust in organic than conventional production (Henneron et al., 2015; Treonis et al., 2018), but results vary by study (Quist et al., 2016; Salas and Achinelly, 2020), suggesting that climate, crop, and production system parameters influence responses. Therefore, in order to validate specific production system impacts, it is important to investigate specific crops and production system parameters. Carrot production in the Southeast – like most vegetable production – is done under uniquely intensive practices due to the high crop value and intense pest pressure in the region. Thus far, most production system research on the nematode community has focused on lower value crops (Ilieva-Makulec et al., 2016; Treonis et al., 2018) or cooler climates with less pest pressure (Quist et al., 2016; Reilly et al., 2013). Among nematode community research in more intensively managed crops – typically tomatoes – in warm climates, fumigation is often not included (Berkelmans et al., 2003; Briar et al., 2011; Sanchez-Moreno et al., 2009), although fumigation is a universal practice in Southeast conventional vegetable production. Because of these differences, production system impacts on the nematode community in prior research may not reflect carrot and vegetable production in the Southeast, so further research is needed to determine production system impacts under those cropping conditions.

In both conventional and organic production, nutrient amendment application rates – whether supplied by conventional fertilizers or organic amendments – are important for both maximizing yield or profitability and minimizing environmental impacts such as nutrient leaching. Nutrient amendments provide resources that not only crops, but also soil-dwelling organisms may utilize. Therefore, nutrient application may affect the soil community, including nematodes (Habteweld et al., 2020; Sarathchandra et al., 2001), and assessing this may provide broader context of their environmental impact. Most nutrient amendment impacts on nematodes are indirect. The flush of resources from nutrient application, particularly carbohydrate sources from organic amendments, can provide resources for the soil microbial community to increase (Reilly et al., 2013; Wolf and Wagner, 2005). In turn, microbe-feeding nematode populations such as fungivores and bacterivores may also increase along with their food source (Grabau et al., 2018; Zhang et al., 2016). In the short term, this flush of resources may favor extreme colonizers or R-strategy adapted organisms that increase rapidly in response to resources (Grabau et al., 2019; Renco and Kovacik, 2012). In contrast, synthetic or mined conventional fertilizers without carbohydrate sources often have a smaller effect on the soil community (Grabau et al., 2018, 2019). Nutrient amendments may also affect the nematode community indirectly through impacts on crop productivity (Bao et al., 2010; Melakeberhan, 2007). Finally, some nutrient amendments may have nematicidal properties (Xiao et al., 2007, 2008).

The impact of nutrient source (manure, compost, or inorganic) on the nematode community has been investigated relatively extensively (Grabau et al., 2019; Zhang et al., 2016), but more research is needed to understand the influence of common grower nutrient rates – a range of positive rates – on the soil community. Evaluating a range of positive nutrient rates most accurately reflects grower practices, and thus potential practical impacts. In most nutrient source studies, the only rate comparison is with an untreated control (Grabau et al., 2018; Wang et al., 2006a). Some nutrient rate studies have been done, but research specific to vegetable systems in a subtropical climate like the Southeast are still needed since responses may vary based on these conditions. Most prior rate studies have been conducted in row crop (Koenning and Barker, 2004; Yang et al., 2016), pasture systems (Gruzdeva et al., 2007; Orwin et al., 2021), or horticultural systems in temperate climates (Forge et al., 2020).

Based on these needs, the objectives of this research were to assess the influence of (i) production system – conventional or organic – on the nematode community, and (ii) nutrient rates on the nematode community in organic and conventional production.

## Materials and methods

### Site and experimental design

To investigate these objectives, trials were conducted at the North Florida Research and Education Center-Suwannee Valley near Live Oak, FL (30.304621, -82.899979). Separate trials were conducted using conventional practices and using organic practices. The organic production system trials were conducted on land certified since 2012 by Quality Certification Services, Gainesville, FL in accordance with the United States Department of Agriculture Organic Standards (United States Code, 2000). As required, all inputs and methods were compliant with USDA National Organic Standards, described in a farming system plan, and approved by the certifying agency. All trials were conducted on deep sand soils typical of the area. For conventional production, the soil was Hurricane-Sandy type (siliceous, Thermic Oxyaquic Alorthod). For organic production, it was a Chipley-Foxworth-Albany soil series complex (Thermic, coated Aquic or Typic Quartzipsamments and Loamy, siliceous, subactive, thermic Aquic Arenic Paleudults). A trial using each production system was conducted in 2016–2017 (Year 1) and again in 2017–2018 (Year 2). Trial locations within the station were moved from Year 1 to Year 2. All locations had a history of spring or fall vegetable production and winter cover crops including rye (*Secale cereale*) in conventional production. In organic production, a mixture of sorghum-sudan grass (*Sorghum* x *drummondii*) and ‘Iron Clay’ cowpea (*Vigna unguiculata*) was grown as a cover crop in summer 2016 and sunn hemp (*Crotalaria juncea*) was grown in summer 2017. Each production system was managed with conventional tillage, which was done frequently for weed management and residue incorporation prior to study establishment.

For each trial, the experiment was a randomized complete block design with four replicates. Each plot was 18 m long and consisted of one bed with 102 cm wide top and 1.3 m from bed center to adjacent bed center. Nitrogen rate treatments were 168, 224, 280, 336, and 392 kg N/ha for both conventional and organic trials. Carrot production without nitrogen fertilizer or amendment is not commercially viable in the Southeast, so a control treatment without nitrogen amendment was not included. Nutrient amendment was applied to the bed tops only, and rates were calculated using the linear bed foot method (Hochmuth and Hanlon, 2012). Nutrient amendment rates were chosen based on the prior guideline of 196 kg N/ha from the University of Florida (Liu et al., 2020). Nitrogen amendment application was distributed throughout the year to match plant nitrogen demand and timing varied by rate and production system (Tables 1 and 2).

**Table 1.** Nutrient amendment application timing by *N* rate treatment in conventional production.

	Year 1 (2016–2017)																
Date	20 Oct	28 Nov	13 Dec	20 Dec	27 Dec	3 Jan	10 Jan	17 Jan	24 Jan	31 Jan	7 Feb	14 Feb	20 Feb	28 Feb	7 Mar	14 Mar	21 Mar
DAP^a^:	**‒**9	30	45	52	59	66	73	80	87	94	101	108	114	122	129	136	143
Treatment (total kg N/ha)	-----------------------------------------N applied each date (kg/ha)--------------------------------------------																
168	28.0	17.5		17.5			17.5		17.5		17.5		17.5		17.5		17.5
224	28.0	24.5		24.5			24.5		24.5		24.5		24.5		24.5		24.5
280	28.0	31.5		31.5			31.5		31.5		31.5		31.5		31.5		31.5
336	28.0	20.5	20.5		20.5	20.5	20.5	20.5	20.5	20.5	20.5	20.5	20.5	20.5	20.5	20.5	20.5
392	28.0	24.3	24.3		24.3	24.3	24.3	24.3	24.3	24.3	24.3	24.3	24.3	24.3	24.3	24.3	24.3
	Year 2 (2017–2018)																
Date:	27 Oct	4 Dec	18 Dec	21 Dec	28 Dec	4 Jan	11 Jan	16 Jan	25 Jan	1 Feb	8 Feb	13 Feb	22 Feb	1 Mar	8 Mar	13 Mar	22 Mar
DAP:	‒6	32	46	49	56	63	70	75	84	91	98	103	112	119	126	131	140
Treatment (total kg N/ha)	-----------------------------------------N applied each date (kg/ha)--------------------------------------------																
168	28.0	17.5		17.5			17.5		17.5		17.5		17.5		17.5		17.5
224	28.0	24.5		24.5			24.5		24.5		24.5		24.5		24.5		24.5
280	28.0	31.5		31.5			31.5		31.5		31.5		31.5		31.5		31.5
336	28.0	20.5	20.5		20.5	20.5	20.5	20.5	20.5	20.5	20.5	20.5	20.5	20.5	20.5	20.5	20.5
392	28.0	24.3	24.3		24.3	24.3	24.3	24.3	24.3	24.3	24.3	24.3	24.3	24.3	24.3	24.3	24.3

**Note:** ^a^DAP is days after planting.

**Table 2.** Nutrient amendment application timing by rate treatment in organic production for both Year 1 and Year 2.

Treatment (total kg N/ha)	At planting^a^	5 WAP^b^	7 WAP
N rate (kg/ha) per timing			
168	84	42	42
224	112	56	56
280	140	70	70
336	168	84	84
392	196	98	98

**Notes:** ^a^In Year 1 (2016), preplant fertilizer was applied on 20 Oct, carrots were planting on 24 Oct and replanted on 14 Nov due to poor stand in initial planting; ^b^WAP is weeks after planting. For each treatment 50% of total was applied at planting with 25% each applied at 5 and 7 WAP.

In conventional production, preplant starter fertilizer was applied to flat ground, rototilled to a depth of 15 cm, and pressed into bare ground beds (10 cm high and 102 cm wide). Starter fertilizer source was 13-1.8-10.8 (N-P-K) and 14-1.8-11.6 in 2017 and 2018, respectively. In-season fertilizer for conventional production was ammonium nitrate (32-0-0) banded on the bed top evenly using a single hopper fertilizer drop spreader with directional spouts (First Products, Tifton, GA). In conventional production, nutrient rates other than N were constant across treatments.

In organic production, all nutrient amendments were approved for use in organic production by the certification agency (Quality Certification Services, Gainesville, FL). For nitrogen rate treatments, poultry litter was used each year. In Year 1, Microstart 60 (Perdue AgriRecycle LLC, Seaford, DE) was used and returned an analysis of 3%-2%-3% (N, P_2_O_5_, K_2_O). In Year 2, locally sourced poultry litter was used and it returned an analysis of 2.9-1.42-3.08. Half of the poultry litter was broadcast and incorporated before planting with the remaining poultry litter applied 5 and 7 weeks after planting (Table 2). Preplant litter was applied evenly by hand to shaped bed tops and lightly incorporated to 4 cm deep with a basket weeder followed by bed re-shaping. Post-plant fertilizer was banded on the bed top between carrot rows and incorporated to 4 cm with a basket weeder. Because axenic phosphorous and potassium fertilizers are not readily available for organic production, PK rates varied along with N rate treatments.

### Trial management

Aside from nutrient rate treatments, each trial was managed uniformly based on standard commercial practices in the area. Specific dates for important maintenance activities are provided in Table 3. For each trial, the cultivar Choctaw was direct seeded in the fall using a sponge-type Seed Spider planter (Sutton Agricultural Enterprises, Inc., Salinas, CA). Seed was planted 0.64 cm deep and the press roller on the planter firmed the soil immediately after seeding. In conventional production, carrots were planted in two sets per bed top, with 30.5 cm between sets. Each set had four rows per bed top spaced 4.76 cm apart, such that there eight rows total on each bed top. In organic production, row spacing was wider with four rows per bed top spaced 17.8 cm apart. Wider row spacing was used to accommodate potential mechanical weeding, although ultimately weeding was done primarily by hand in the organic production system. Carrots were harvested mechanically in spring (Table 3) with yield data and other relevant production results forthcoming in separate reports (unpubl. data).

**Table 3.** Schedule for data collection and trial establishment.

	Year 1 (2016–2017)		Year 2 (2017–2018)	
Item	Conventional	Organic	Conventional	Organic
Soil fumigation	9 Sep (‒50)	N/A^a^	13 Oct (‒21)	N/A
Date planted	29 Oct (0)	14 Nov (0)^b^	2 Nov (0)	15 Nov (0)
Date harvested	10–11 Apr (170)	17 Apr (161)	19–20 Apr (168)	20 Apr (155)
Midseason soil sampling	16 Dec (48)	6 Jan (53)	2 Jan (61)	2 Jan (48)
Harvest soil sampling	28 Mar (157)	28 Mar (141)	13 Mar (131)	13 Mar (118)

**Notes:** Numbers in parentheses are days before transplanting (DBP) or days after transplanting (DAP). ^a^In organic production, *Purpureocillium liacinum* was applied for nematode biocontrol; ^b^In Year 1 organic production, carrots were initially planted on 24 Oct, but replanted due to poor stand.

In conventional production, the entire trial was fumigated for plant-parasitic nematode management before planting using 1,3-dichloropropene (Telone II, Dow Agrosciences, Wilmington, DE) at 168 L/ha. Fumigation was conducted using a broadcast shank rig with 30 cm spacing between shanks and fumigant released approximately 30–35.6 cm deep in the soil profile. In organic production, for nematode control, a commercial formulation of live *Purpureocillium lilacinum* fungi (Melocon WG, Certis USA LLC, Columbia, MD) was used for nematode biocontrol. The *P. lilacinum* formulation was applied to the soil using a CO_2_-powered backpack sprayer at labelled rates on 19 October, 12 January, and 27 January (‒26, 59, and 74 DAP, respectively) in Year 1. In Year 2, *P. lilacinum* was applied on 1 December and 1 February (16 and 78 DAP).

Supplemental fertilizer applications were made in both organic and conventional systems and were uniform across N rate treatments. In both systems, dolomitic lime and boron were broadcast preplant each season at 2,242 and 1.12 kg/ha, respectively. In conventional production, 112 kg/ha potash was applied in the form of two midseason broadcast applications of Sul-Po-Mag (0N-0P-18.3K-22S-11Mg) per season. In conventional production, phosphate was applied at 52 kg/ha in Year 2, but not Year 1.

In-season weed management was accomplished primarily by hand weeding in organic production. In conventional production, weeds were well-managed with two post-emergence applications of linuron chemical herbicide. *Alternaria* leaf blight was the main disease of concern in both production systems. In both systems, this disease was managed using weekly fungicide applications when weather was wet and conducive to this pathogen. In conventional production, a variety of chemical fungicides were applied, rotating chemistries by FRAC (Fungicide Resistance Action Committee) code to minimize pathogen resistance. In organic production, organic compliant fungicides were used including *Strepomyces* bacteria (Actinovate AG, Valent BioSciences LLC, Walnut Creek, CA) and copper (Nordox 75WG, Nordox, Oslo, Norway) in Year 1. In Year 2, copper and a hydrogen peroxide-peroxyacetic acid formulation (OxiDate 2.0, BioSafe Systems, LLC., East Hartford, CT) were rotated. In both production systems, cereal rye cover crop was grown in trial alleys to protect plants from blowing sand and serve as a barrier to wind-borne pathogens.

### Nematode quantification

Nematode soil populations in each plot were quantified at midseason and near harvest each year. Using an Oakfield tube, 12 soil cores to 25 cm depth were collected from the carrot rooting zone. Soil was homogenized manually, and nematodes were extracted using the sucrose-centrifugation method (Jenkins, 1964). The nematode community (plant-parasitic and free-living nematodes) were quantified morphologically to genera level by microscope.

Following nematode community quantification, abundances of individual trophic groups – including bacterivores, fungivores, herbivores, and omnivore-predators – were calculated based on published groupings for individual nematode families (Yeates et al., 1993). The most abundant genera (Table 4) were also subjected to analysis. This included bacterivores *Cephalobus* spp. and *Rhabditis* spp., fungivores *Aphelenchus* spp. and *Aphelenchoides* spp., and plant-parasitic nematodes *Mesocriconema* spp. and *Meloidogyne incognita*. *Paratylenchus* spp. was also abundant at harvest 2017 in the organic system (228 nematodes/100 cm^3^ soil), but was not included in analysis as it was not abundant in any other season-system.

**Table 4.** Nematode genera in organic and conventional trials.

			-----------Organic----------		-------Conventional---------	
Nematode genera	Feeding group	cp value	Relative abundance^a^	Mean abundance^b^	Relative abundance	Mean abundance
*Rhabditis*	Bacterivore	1	25.95%	506	14.38%	79
*Panagrolaimus*	Bacterivore	1	1.67%	33	0.03%	0
*Diplogaster*	Bacterivore	1	0.04%	1	0.14%	1
*Cephalobus*	Bacterivore	2	11.27%	220	56.71%	310
*Acrobeles*	Bacterivore	2	9.31%	182	1.07%	6
*Eucephalobus*	Bacterivore	2	9.08%	177	7.49%	41
*Cervidellus*	Bacterivore	2	0.74%	14	0.08%	0
*Heterocephalobus*	Bacterivore	2	0.15%	3	N/A	N/A
*Plectus*	Bacterivore	2	0.12%	2	0.31%	2
*Filenchus*	Fungivore	2	5.91%	115	0.77%	4
*Aphelenchus*	Fungivore	2	3.92%	77	6.21%	34
*Aphelenchoides*	Fungivore	2	3.26%	64	5.00%	27
*Ditylenchus*	Fungivore	2	0.36%	7	0.07%	0
*Diphtherophora*	Fungivore	3	0.23%	4	0.10%	1
*Paratylenchus*	Herbivore	2	3.21%	63	0.08%	0
*Malenchus*	Herbivore	2	0.89%	17	0.02%	0
*Mesocriconema*	Herbivore	3	11.45%	223	4.62%	25
*Pratylenchus*	Herbivore	3	6.52%	127	0.24%	1
*Meloidogyne*	Herbivore	3	0.18%	4	0.82%	4
*Paratrichodorus*	Herbivore	4	0.23%	5	0.28%	2
*Axonchium*	Herbivore	5	0.10%	2	0.16%	1
*Thonus*	Omnivore	4	1.48%	29	0.36%	2
*Mesodorylaimus*	Omnivore	4	0.36%	7	N/A	N/A
*Eudorylaimus*	Omnivore	4	0.14%	3	0.04%	0
*Aporcelaimellus*	Omnivore	5	2.94%	57	0.33%	2
*Paraxonchium*	Omnivore	5	0.04%	1	N/A	N/A
*Seinura*	Predator	2	N/A	N/A	0.50%	3
*Discolaimus*	Predator	5	0.13%	3	0.02%	0

**Notes:** Data combined across sampling dates and years. ^a^Relative abundance is proportion of total nematode abundance across all sampling dates and years within production system. Only nematode genera with 0.1% relative abundance in either organic or conventional production are shown; ^b^Mean abundance is mean nematodes/100 cm^3^ soil across all sampling dates and years within production system.

Key nematode community indices were also calculated based on nematode abundances. Maturity index, structure index, enrichment index, channel index, and Hills N1 diversity were calculated. Briefly, the maturity index is a measure of system disturbance based on the average nematode colonizer-persister (cp) value in a sample (Bongers, 1990; Bongers and Bongers, 1998). The cp value is a 1–5 ranking of the life strategy of nematodes with 1 indicating an extreme colonizer (short life cycle, short generation time, and high reproductive capacity) and 5 indicating an extreme persister (longer life cycle, longer generation time, and low reproductive capacity). The structure index is a measure of the number of trophic links in a system based on the abundance of extreme colonizers relative to nematodes common in most environments – namely cp2 fungivores and bacterivores (Ferris et al., 2001). The enrichment index is a measure of resource enrichment in a system based on relative abundances of enrichment opportunists (Ferris et al., 2001). The channel index is a measure of decomposition pathways with higher values indicating predominantly fungal decomposition channels and lower values indicating bacterial decomposition channels (Ferris et al., 2001). Hills N1 diversity was calculated using either nematode genera or nematode guilds – trophic group and cp value combinations such as cp2 bacterivores. Hills N1 diversity is derived from the Shannon diversity index and values are interpreted as a measure of the number of common genera or guilds in a sample (Neher and Darby, 2006). Trophic guild abundances were calculated, but not included in analyses due to similarity to either the most abundant genera in a particular guild or total abundance for a particular trophic group.

### Statistical analysis

Variables were analyzed separately for each sampling date. Initially, data were subject to one-way ANOVA for combining experiments. In that analysis, year was treated as a random effect, not of scientific interest, whereas production system was treated as a fixed effect of interest. Effect of production system was determined using year by production system interaction as the error term (Carmer et al., 1989). Due to significant interactions, nutrient rate effects were subsequently analyzed separately by production system as well as date. Before completing ANOVA, response variables were transformed, if needed, to meet assumptions of homogeneity of variance using Levene’s test (Levene, 1960) and normality of residuals based on graphing (Cook and Weisburg, 1999). For combining experiments ANOVA, all nematode populations were transformed by natural log(*x*+1) for both midseason and harvest. Additionally, guild and genera diversity were transformed by *x*^2 at midseason for combining experiment analysis. No other variables were transformed at midseason or harvest for combining experiments analysis. For one-way ANOVA within production system, bacterivore abundances were transformed by natural log(*x*+1) for conventional production midseason Year 1 and organic production Year 1 midseason and harvest. *Rhabditis* spp. abundance was square-root transformed for organic Year 1 harvest. *Aphelenchoides* spp. abundance was transformed by *x*^^(3/2)^ for organic harvest Year 2 and conventional production midseason Year 1 and harvest Year 2. *Cephalobus* spp. populations were transformed by natural log(*x*+1) for conventional production midseason Year 1. Variables for all other analyses were not transformed. Production system effects were considered significant at *α*=0.05 in ANOVA. For nutrient rate treatment, means were separated by Fisher’s protected LSD (*α*=0.05) if main effects were significant in ANOVA. Analyses were conducted in R statistical software (version 3.4.4, The R Foundation for Statistical Computing, Vienna, Austria).

## Results

### Production system impacts on the nematode community

**Figure 1:**
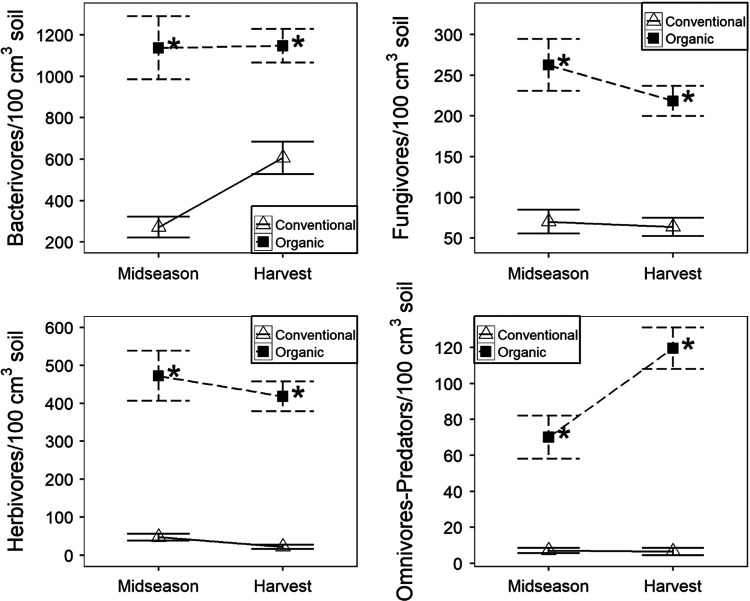
Nematode soil abundances for different trophic groups as affected by production system. Data are combined across two years. Data points and error bars represent means and standard errors, respectively. ‘Conventional’ and ‘organic’ are conventional and organic production systems, respectively. An ‘*’ beside organic production mean indicates significant production system effects for a given season (ANOVA, *p* < 0.05).

**Figure 2:**
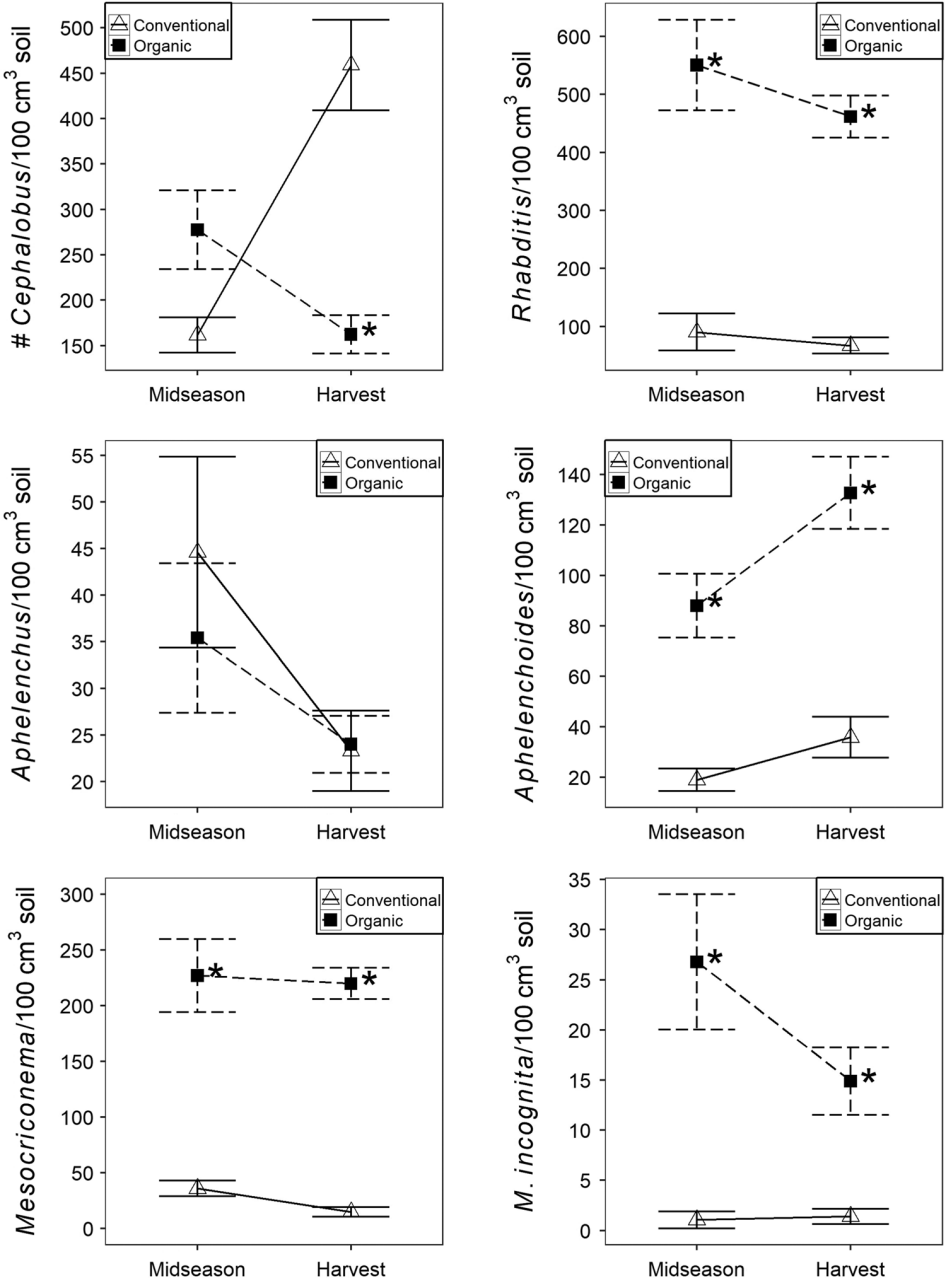
Nematode soil abundances for common genera as affected by production system. Data are combined across two years. Data points and error bars represent means and standard errors, respectively. ‘Conventional’ and ‘organic’ are conventional and organic production systems, respectively. An ‘*’ beside organic production mean indicates significant production system effects for a given season (ANOVA, *p* < 0.05).

The nematode community was consistently different between conventional and organic production systems. Soil abundances of each free-living nematode trophic group – bacterivores, fungivores, and omnivore-predators – as well as herbivores were significantly greater in organic than conventional production at both midseason and harvest (Fig. 1). Sensitivity did vary by genera as *Rhabditis* spp. (cp1 bacterivore), *Aphelenchoides* spp. (cp2 fungivore), *Mesocriconema* spp. (minor plant-parasite), and *M. incognita* (major plant-parasite) were greater in organic than conventional production at both midseason and harvest (Fig. 2). *Cephalobus* spp. (cp2 bacterivore) was not affected by production system at midseason and was greater in conventional than organic production at harvest. *Aphelenchus* spp. (cp2 fungivore) was not affected by production system.

**Figure 3:**
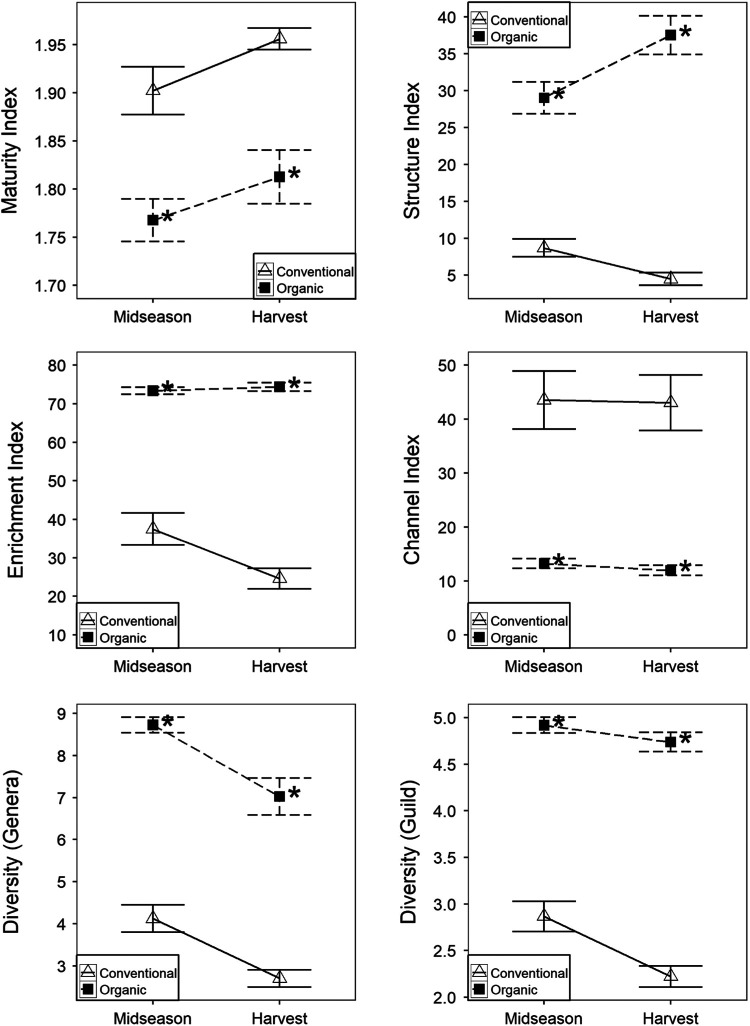
Nematode community indices as affected by production system. Data are combined across two years. Data points and error bars represent means and standard errors, respectively. ‘Conventional’ and ‘organic’ are conventional and organic production systems, respectively. An ‘*’ beside organic production mean indicates significant production system effects for a given season (ANOVA, *p* < 0.05). Diversity is Hill’s N1 diversity based on nematode genera or trophic guilds.

Similarly, all nematode community indices were affected by production system at both midseason and harvest. The maturity and channel indices were each decreased in organic relative to conventional production (Fig. 3). The enrichment index, structure index, and Hills N1 diversity – derived from both nematode genera and nematode trophic guilds – were each decreased in conventional production relative to organic production.

### Nutrient rate impacts on the nematode community

Nutrient rate impacts on nematode abundances were relatively inconsistent, and bacterivores and fungivores were the groups most often affected. In organic production, bacterivore and fungivore abundances were greater at higher nutrient rates in Year 1, but unaffected in Year 2 (Table 5). Overall bacterivore abundances in organic production were greater at 336 kg N/ha than certain lower rates at midseason and harvest Year 1, but were unaffected in either season in Year 2 (Table 5). This was driven primarily by extreme enrichment opportunists as *Rhabditis* spp. (cp1 bacterivore) had similar trends to overall bacterivores. In contrast, the more basal enrichment opportunist *Cephalobus* spp. (cp2 bacterivore) was not significantly affected by nutrient rate in organic production (Table 5). At harvest in Year 1 in organic production, fungivore abundances were greater at 392 kg N/ha than 224 or 280 kg N/ha, but were not affected at any other date. Both common fungivore genera (*Aphelenchus* spp. and *Aphelenchoides* spp.) followed similar trends to overall fungivore abundances in organic production, although *Aphelenchoides* spp. was affected at both midseason and harvest of Year 1 whereas *Aphelenchus* spp. was only affected at harvest Year 1.

**Table 5.** Organic production: nematode soil abundances for bacterivores and fungivores (total and individual genera) as affected by nitrogen rate^a^.

Year 1			Year 2		Year 1		Year 2	
Rate (kg N/ha)	Pm^b^	Pf	Pm	Pf	Pm	Pf	Pm	Pf
Bacterivores					Fungivores			
168	192 b	719 b	1,615	1,246	89	144 bc	452	327
224	223 b	768 b	1,939	1,150	79	101 c	389	191
280	259 ab	703 b	1,980	1,274	83	108 c	355	248
336	360 a	1877 a	2,396	1,186	116	229 ab	565	226
392	305 ab	1,124 ab	2,109	1,431	106	276 a	393	335
*Rhabditis* spp.					*Aphelenchus* spp.			
168	97 b	362 b	757	413	7	23 b	67	21
224	100 ab	361 b	899	466	6	24 b	55	0
280	113 b	251 b	1,017	450	8	17 b	45	31
336	193 a	872 a	1,182	428	11	36 ab	48	10
392	165 ab	487 ab	984	526	13	49 a	96	30
*Cephalobus* spp.					*Aphelenchoides* spp.			
168	25	38	468	295	33 b	99 bc	102	187
224	41	95	439	189	44 ab	61 c	108	90
280	54	31	534	331	36 b	87 bc	121	139
336	58	87	555	269	61 a	184 ab	207	89
392	33	97	570	190	51 ab	214 a	118	178

**Notes:** ^a^Values are mean nematode soil abundances (nematodes/100 cm^3^ soil); ^b^Pm and Pf indicate midseason and final nematode soil abundances, respectively. Values for the same variable with different letters in the same column are significantly different according to Fisher’s protected LSD (*p* < 0.05). Absence of letters indicates there were no significant nutrient rate effects in that season for a particular variable (ANOVA, *p* > 0.05).

Conventional nutrient rates had inconsistent effects on fungivores and bacterivores (Table 6). At harvest Year 1, greater nutrient rates (336 and 392 kg N/ha) increased total bacterivores and *Cephalobus* spp. relative to the lower rate of 224 kg N/ha. In contrast, in Year 2 at harvest, a moderate nutrient rate (224 kg N/ha) had the greatest total bacterivore abundance, but *Cephalobus* spp. was not affected by conventional nutrient rates in Year 2. *Rhabditis* spp. was not significantly affected by conventional nutrient rate. Moderate nutrient rates (224 or 280 kg N/ha in Year 2 or Year 1, respectively) increased total fungivore abundances in Year 1 and Year 2 harvest relative to extreme rates. *Aphelenchoides* spp. followed a similar trend in Year 2, but *Aphelenchus* spp. was unaffected in either year. Omnivore-predators were unaffected by nutrient rate in either organic or conventional production in any season (data not shown).

**Table 6.** Conventional production: nematode soil abundances for bacterivores and fungivores (total and individual genera) as affected by nitrogen rate^a^.

Year 1			Year 2		Year 1		Year 2	
Rate (kg N/ha)	Pm^b^	Pf	Pm	Pf	Pm	Pf	Pm	Pf
Bacterivores					Fungivores			
168	95	220 ab	351	756 b	10	15 b	94	106 b
224	119	156 b	706	1,563 a	19	15 b	203	194 a
280	89	216 ab	542	967 b	7	29 a	148	93 b
336	93	273 a	356	770 b	10	10 b	140	111 b
392	84	316 a	280	829 b	7	13 b	61	51 b
*Rhabditis* spp.					*Aphelenchus* spp.			
168	2	2	131	73	9	7	70	42
224	2	2	359	214	14	8	138	56
280	4	2	240	149	6	14	99	46
336	1	3	103	116	8	7	53	23
392	2	3	60	108	5	6	45	26
*Cephalobus* spp.					*Aphelenchoides* spp.			
168	89	214 ab	186	623	1	4	20	62 b
224	115	152 b	295	933	4	5	52	134 a
280	82	211 ab	277	695	1	10	43	46 b
336	89	267 a	210	587	0	1	53	69 ab
392	80	311 a	196	597	1	5	15	23 b

**Notes:** ^a^Values are mean nematode soil abundances (nematodes/100 cm^3^ soil); ^**b**^Pm and Pf indicate midseason and final nematode soil abundances, respectively. Values for the same variable with different letters in the same column are significantly different according to Fisher’s protected LSD (*p* < 0.05). Absence of letters indicates there were no significant nutrient rate effects in that season for a particular variable (ANOVA, *p* > 0.05).

Total herbivores and *Mesocriconema* spp. were not significantly affected by nutrient rate in either conventional or organic production in any season (Table 7). Soil abundances of *M. incognita* were significantly greater for a moderate nutrient rate (280 kg N/ha) than extreme rates in midseason of Year 2 of organic production. For Year 1 of organic production and both years of conventional production, *M. incognita* soil abundances were relatively low and unaffected by nutrient amendment application.

**Table 7.** Herbivore and herbivore genera soil abundances as affected by nitrogen rate in organic and conventional production^a^.

Organic production					Conventional production			
Year 1			Year 2		Year 1		Year 2	
Rate (kg N/ha)	Pm^b^	Pf	Pm	Pf	Pm	Pf	Pm	Pf
Herbivores								
168	80	648	860	260	13	13	80	648
224	87	567	804	359	7	4	87	567
280	85	310	821	312	7	5	85	310
336	92	511	950	367	4	3	92	511
392	75	500	872	350	5	3	75	500
*Mesocriconema*								
168	48	258	342	187	10	10	48	258
224	49	243	332	291	3	2	49	243
280	65	194	436	194	4	3	65	194
336	58	212	446	221	4	1	58	212
392	50	211	444	187	4	2	50	211
*Meloidogyne incognita*								
168	1	0	29 b	18	0	0	1	0
224	2	3	41 b	28	0	0	2	3
280	0	6	98 a	18	0	0	0	6
336	0	0	49 b	43	0	0	0	0
392	0	8	49 b	25	0	0	0	8

**Notes:** ^a^Values are mean nematode soil abundances (nematodes/100 cm^3^ soil); ^b^Pm and Pf indicate midseason and final nematode soil abundances, respectively. Values for the same variable with different letters in the same column are significantly different according to Fisher’s protected LSD (*p* < 0.05). Absence of letters indicates there were no significant nutrient rate effects in that season for a particular variable (ANOVA, *p *> 0.05).

Nutrient rates had minimal effect on nematode community indices in either conventional or organic production. In midseason Year 1 of organic production (Table 8), the enrichment index was greater at high nutrient rates (336 or 392 kg N/ha) than a lower rate (224 kg N/ha). There were no significant nutrient rate effects on structure index, or Hill’s N1 diversity based on either genera or guild (Table 8). Similarly, maturity index and channel index were not significantly affected by nutrient rate in any season or production system (data not shown).

**Table 8.** Nematode community indices as affected by nitrogen rate in organic and conventional production^a^.

Organic production					Conventional production			
Year 1			Year 2		Year 1		Year 2	
Rate (kg N/ha)	Vm^b^	Vf	Vm	Vf	Vm	Vf	Vm	Vf
Structure index								
168	37	50	27	25	8	5	10	1.5
224	26	50	36	37	7	5	6	6.6
280	34	48	37	28	7	6	7	9.4
336	28	46	29	24	11	3	16	4.4
392	20	37	18	33	8	3	6	0.3
Enrichment index								
168	72 ab	76	73	73	13	10	65	35
224	70 b	71	72	73	18	11	61	42
280	70 ab	69	77	72	19	13	66	41
336	76 a	81	74	76	10	6	59	43
392	75 a	76	74	76	12	8	54	37
Hill’s N1 diversity-genera								
168	9.4	9.5	8.6	4.6	2.5	2.1	6.2	3.1
224	9.4	9.8	9.1	4.4	3.0	1.9	5.4	4.0
280	8.8	9.7	8.5	4.5	2.4	2.1	5.2	4.0
336	8.0	8.8	8.9	4.6	2.3	1.4	7.1	4.0
392	8.1	9.8	8.4	4.5	2.3	1.6	4.9	2.8
Hill’s N1 diversity-guild								
168	2.0	1.8	4.1	2.8	5.1	5.1	5.3	4.5
224	2.2	1.6	3.4	3.0	4.8	5.2	5.3	4.6
280	2.1	1.8	3.6	2.9	4.8	4.8	5.2	4.4
336	1.9	1.3	4.2	2.9	4.5	4.6	5.0	4.5
392	1.9	1.5	3.4	2.6	4.4	5.1	4.8	4.8

**Notes:** ^a^Values are mean nematode soil abundances (nematodes/100 cm^3^ soil); ^b^Vm and Vf indicate midseason and final values, respectively. Values for the same variable with different letters in the same column are significantly different according to Fisher’s protected LSD (*p* < 0.05). Absence of letters indicates there were no significant nutrient rate effects in that season for a particular variable (ANOVA, *p* > 0.05).

## Discussion

Organic production clearly stimulated a more abundant and diverse nematode community than conventional production. Ostensibly, enhancing the free-living nematode community also reflects enhancing the overall activity and function of the soil community in organic compared with conventional production and could be a considered a contribution to promoting ecological balance and biodiversity, stated purposes of organic production. In prior research, organic production often enhances the nematode community relative to conventional production, but impacts are not usually as broad across trophic groups as in this study (Salas and Achinelly, 2020; Sanchez-Moreno et al., 2009; Treonis et al., 2018). Organic production usually increases only one or two trophic groups or genera, predominantly bacterivores (Briar et al., 2007; Ferris et al., 1996; Henneron et al., 2015; Overstreet et al., 2010), sometimes omnivore-predators (Henneron et al., 2015; Quist et al., 2016), and more rarely plant-parasites (Li et al., 2014) or fungivores (Treonis et al., 2018).

Much of this variation among studies is likely due to the different practices that define production systems in each study based on standards for that crop and location. For this study, fumigation using 1,3-D in the conventional system was likely a very influential system component as it often broadly decreases nematode community populations in factorial studies (Grabau et al., 2020; Timper et al., 2012; Watson and Desaeger, 2019). In contrast, no fumigation was used in organic production and the biological nematicide applied, live *P. lilacinus* fungi, is likely to both have fewer non-target, broad-spectrum effects and be less effective at managing plant-parasitic nematodes than fumigation (Baidoo et al., 2017; Crow, 2013). Most prior studies, even in intensive systems, did not include fumigation (Berkelmans et al., 2003; Briar et al., 2011; Henneron et al., 2015; Sanchez-Moreno et al., 2009), which likely contributed to production system impacts being less broad spectrum in those studies. In contrast, nutrient source effects may have been a more important factor in other production system studies. Organic nutrient amendment is known to affect primarily bacterivore and enrichment opportunist nematodes (Grabau et al., 2018, 2019; Zhang et al., 2016), and nutrient source has been identified as a major factor driving differences among production systems (Briar et al., 2007; Ferris et al., 1996; Henneron et al., 2015; Overstreet et al., 2010). While individual management components are likely to have a strong role in production system effects, it must also be emphasized that the set of components that defines a system determine its impacts.

Whereas enhancing free-living nematode communities was a potentially beneficial impact of organic production, increasing plant-parasitic nematode pressure was a negative consequence of organic production. Plant-parasitic nematode impacts on production were likely not substantial in this study as the only serious pest, *M. incognita*, was generally not present in high abundances in either production system. The presence of *M. incognita* does accurately reflect increased challenges of pest management in organic production, particularly in the southeastern United States and other environments conducive to pest pressure. Because available organic nematicides are generally not as effective as fumigation (Desaeger and Watson, 2019; Watson and Desaeger, 2019), an integrated approach also utilizing crop rotation, cover cropping, and resistant cultivars for available crops is important in organic production. Organic growers generally have a longer time between horticulture crops in their rotation than was feasible on a research station, which is one limitation of this study.

Greater nutrient rate rates tended to stimulate enrichment opportunists – bacterivores and fungivores – in both organic and conventional production, although this was not consistent in time, and the responses varied by system. In organic production, extreme enrichment opportunists, namely *Rhabditis* spp., were more responsive to nutrient rates than moderate opportunists such as *Cephalobus* spp., whereas the latter was more responsive in conventional production. Fungivores were also somewhat more responsive to high nutrient rate in organic production than conventional production.

In general, these trends are similar to the limited number of prior nutrient rate studies, and responses to organic nutrient rates are often stronger and more consistent in other studies. One reason for a moderate study response in this study is that nutrient was applied throughout the year, whereas in other studies, particularly with row crops, all or most of the nutrient was applied before planting. In this study, the poultry litter supply source varied by year and the litter nutrient analysis also varied slightly. This could have contributed to year-to-year inconsistency in nutrient rate effects in organic production. Varying crop, nutrient source, climate, and soil type likewise may have influenced response amplitude and consistency among studies. For example, in peanut (*Arachis hypogaea*) production, increased swine manure rates increased bacterivores with cp2 bacterivores more responsive than cp1 (Yang et al., 2016). High poultry litter rates increased bacterivores at midseason and fungivores at harvest in a cotton study (Koenning and Barker, 2004).

In contrast, increasing inorganic nutrient rates had minimal impact on bacterivores or fungivores in blueberry (Forge et al., 2020), pasture (Sarathchandra et al., 2001), or corn (Mashavakure et al., 2018). Similarly, in nutrient source studies, bacterivores and fungivores are typically more responsive to organic amendments than conventional fertilizers (Grabau et al., 2018, 2019; Wang et al., 2006b). The presence of carbohydrate food sources for soil-dwelling organisms in organic, but not conventional fertilizer (Wolf and Wagner, 2005) is likely the main driver for these differences. Duration and repetition of nutrient amendment application may also affect responses as nematode community responses to inorganic fertilizer rates were most substantial five years into a nine-year pasture study (Gruzdeva et al., 2007). Only short-term responses were measured in this study.

Organic and conventional nutrient rates had minimal impact on nematode community diversity or structure based on nematode community indices and populations of higher trophic groups – omnivores and predators. In prior research, higher organic nutrient rates – relative to lower organic nutrient rates, untreated or similar nutrient rates from conventional fertilizer – often decrease community diversity and structure because enrichment opportunists dominate the community, at least in the short term (Grabau et al., 2018; Yang et al., 2016; Zhang et al., 2016). This is in part because both organic (Grabau et al., 2018; Koenning and Barker, 2004) and conventional nutrient amendments (Forge et al., 2020; Sarathchandra et al., 2001) often have minimal impact on omnivores and predators.

In some cases, organic nutrient amendments have increased omnivore or predator populations (Wang et al., 2006b; Yang et al., 2016), likely due to bottom-up effects. Time may be a factor for bottom-up nutrient amendment effects to influence higher trophic groups as suggested in a long-term conventional fertilizer pasture study (Gruzdeva et al., 2007). Omnivores and predators also often have relatively low abundances in agricultural systems (Forge et al., 2020; Grabau et al., 2018), particularly those with more physical or chemical disturbance, which makes it more difficult to detect nutrient amendment effects. Omnivore-predators were relatively abundant in organic production in this study, but still unresponsive to nutrient rate, which may be due, in part, to the short-term nature of this study. Nutrient rate had minimal impacts on herbivore populations in either conventional or organic production. Impacts of nutrient rate on plant-parasitic nematodes were variable in prior studies (Forge et al., 2020; Koenning and Barker, 2004; Wang et al., 2006b), suggesting this interaction is specific to the nutrient source, nematode species, and cropping system.

In practice, nematode community responses to production system or nutrient rates are not likely to drive grower decisions at this time. Economic and yield responses are more important and direct measurements, but this study provides a better understanding of how production choices impact the agricultural environment, namely the soil community. The soil community does influence soil health, and thus crop yield, although likely in a longer-term way that is not yet easy to quantify. Particularly, for production system impacts, continued research in Southeast vegetable production will help validate system-level impacts on the soil community because this study is of limited geographic scope.

In summary, production system has a strong impact on the nematode community in carrot production with organic systems supporting a more abundant and diverse free-living nematode community, but also harboring more plant-parasitic nematodes. In the short-term, increasing nutrient rate increases enrichment opportunist nematodes, but inconsistently, and has minimal impact on higher trophic groups or plant-parasitic nematodes. Both production system and nutrient rate influence the soil community, but production system has a stronger, more consistent impact.
